# A case of IgG4-related tubulointerstitial nephritis concurrent with Henoch-Schönlein purpura nephritis

**DOI:** 10.1186/1710-1492-7-5

**Published:** 2011-03-31

**Authors:** Rukako Tamai, Yoshiyuki Hasegawa, Satoshi Hisano, Katsuhisa Miyake, Hitoshi Nakashima, Takao Saito

**Affiliations:** 1 Department of Internal Medicine, Saiseikai Futsukaichi Hospital, Yumachi 3-13-1, Chikushino city, 818-8516, Japan; 2 Department of Pathology, Faculty of Medicine, Fukuoka University, Nanakuma7-45-1, Johnan-ku, Fukuoka city, 814-0180, Japan; 3 Division of Nephrology and Rheumatology, Department of Internal Medicine, Faculty of Medicine, Fukuoka University, Nanakuma7-45-1, Johnan-ku, Fukuoka city, 814-0180, Japan

## Abstract

We describe a 72-year-old man, who had been suffered from Henoch-Schönlein purpura (HSP) several times, presented with hematoproteinuria with granular cast, and general lymphadenopathy. The immunological examination of the serum showed polyclonal hypergammagloburinemia with high value of IgG4. The renal biopsy revealed interstitial inflammatory cell infiltration, including infiltration of lymphocytes and plasma cells, and segmental glomerulonephritis. Direct immunofluorescence microscopy revealed apparent positive staining with anti-human IgA, and anti-human IgG in glomeruli, anti-human IgG4 antibody staining showed many positive plasma cells in the interstitium. The patient was diagnosed with HSP nephritis that was complicated by IgG4-related nephropathy. As a result of the treatment with 30mg prednisolone, the swelling of the LNs decreased, but the patient continued to have persistent hematoproteinuria.

## Introduction

A novel clinicopathological entity of IgG4-related autoimmune disease characterized by extensive IgG4-positive plasma cell infiltration of organs together with CD4- or CD8-positive T lymphocytes is proposed [1]. Renal involvement in this entity was also suggested, and three patterns of renal involvement have been described: 1) extraparenchymal involvement such as hydronephrosis associated with retroperitoneal lesions; 2) diffuse tubulointerstitial nephritis (TIN); and 3) renal lesions composed of focal lymphoplasmacytic infiltration of the renal interstitium [2]. In this report we describe a rare case diagnosed with HSP nephritis that was complicated by IgG4-related nephropathy.

## Case report

**Figure 1:**
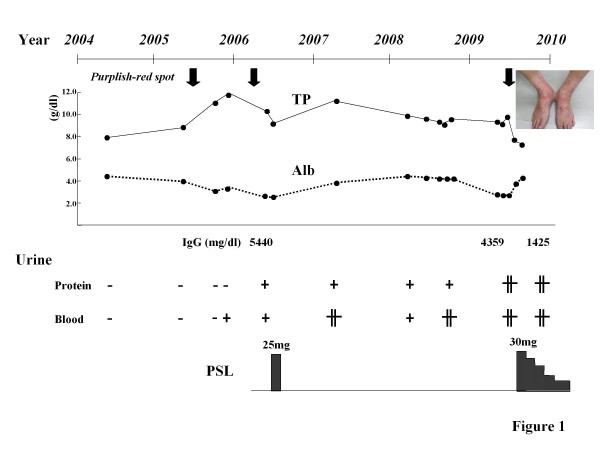
**Clinical course of the patient**. Purplish-red spot in the lower extremities as a picture had been developed 3 times in 6 years (downward bald arrow). Hematoproteinuria has been detected since 2006. TP; serum total protein, Alb; serum albumin, PSL; prednisolone.

A 72-year-old man presented with cervical, axillary, left subclavian, and inguinal lymph nodes (LNs) swelling. The LNs gradually increased in size for 1 month. During this period, the patient often had a low-grade fever and arthralgia. He also experienced a marked weight loss of 7 kg in 3 months. In June 2009, he developed an erythematous rash predominantly on his lower legs and was admitted to the hospital. In 2005, he had developed similar erythematous rashes in the lower extremities several times. In 2006, the patient was diagnosed with Henoch-Schönlein purpura (HSP) on the basis of histological examination of skin biopsy samples, which showed leukocytoclastic vasculitis. Immunohistochemical study with anti-IgA antibody was not performed. A treatment with prednisolone (PSL; 25 mg) had been effective (Figure 1). He had no history of allergic diseases such as bronchial asthma, atopic dermatitis, and allergic rhinitis. In 2002, he underwent gastrectomy for gastric cancer.

**Figure 2:**
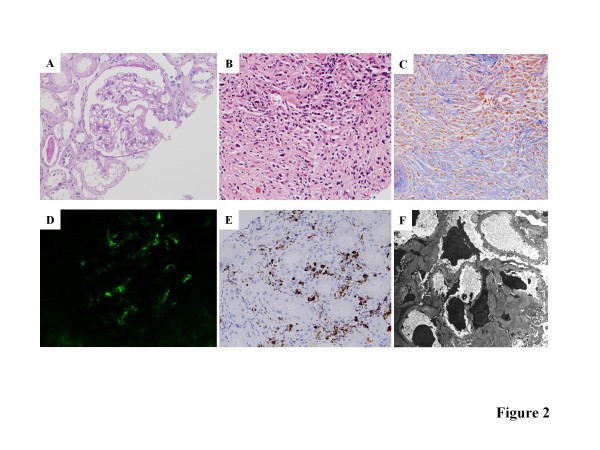
**Representative images of the renal biopsy samples**. **A**. Mild mesangial proliferation is observed. (PAS ×200) **B**. Obvious inflammatory cell infiltration including lymphocytes, plasma cells and eosinophils are found in the interstitium. (PAS ×200) **C**. Massive fibrosis and inflammatory cell infiltration are observed in the interstitium. (Masson Trichrome staining ×200) **D**. Immunofluorescence photomicrographs showing IgA (×400). **E**. Immunostaining reveals abundant IgG4-positive plasma cells in the interstitium. **F**. Electron micrograph shows numerous electron-dense deposits in the mesangium. ×3,000.

On admission, he was febrile, and the rash was palpable and purpuric in nature. A physical examination showed no abnormalities in the lungs, heart, abdomen, and central nervous system. Laboratory findings showed an increased erythrocyte sedimentation rate (73 mm/h) and the value of C-reactive protein was 0.22 mg/dL. The hemoglobin concentration was 11.0 g/dL, the white blood cell count was 8,900/mm^3 ^(neutrophils 66.8%, lymphocytes 21.5%, monocytes 4.1%, eosinophils 7.0%, and basophils 0.6%), and the platelet count was 45.1 × 10^4^/mm^3^. Hematuria and proteinuria with granular cast were detected. The results of the serum chemistry analyses are as follows: serum creatinine, 0.96 mg/dL (normal, 0.4-1.2 mg/dL); blood urea nitrogen, 16.7 mg/dL; total serum protein 8.6 mg/dL (normal, 6.5-8.2 g/dL); and serum albumin 3.6 g/dL (normal, 3.7-5.2 g/dL). Serum transaminase, amylase, and lactate dehydrogenase (LDH) levels were within normal limits. The immunological tests were positive for antinuclear antibody at a titer of 80 dil, and the immunofluorescence patterns were speckled and homogeneous. Anti-double-stranded DNA antibody, rheumatoid factor, anti-Sjögren's syndrome A (anti-SS-A), anti-SS-B antibodies, anti-Sm antibody, anti-Jo-1 antibody, and anti-RNP antibody were all absent. The serum level of immunoglobulin G (IgG) was abnormally high, but IgA and IgM were within normal limits (4,359 mg/dL, 242 mg/dL, and 64 mg/dL, respectively). The serum IgE level was elevated (537 U/mL). Molecules of the subclass IgG4 accounted for 25% (1,100 mg/dL) of the IgG molecules. Serum protein electrophoresis revealed polyclonal hypergammaglobulinemia. Serum levels of C3, C4, and total complement hemolytic activity (CH50) were 55 mg/dL (normal, 86-160 mg/dL), 3 mg/dL (normal, 17-45 mg/dL), and less than 12.0 U/mL (normal, 25-48 U/mL), respectively. Myeloperoxidase antineutrophil-cytoplasmic antibody (MPO-ANCA) was detected at a titer 22 EU (normal, <10EU), but proteinase-3 antineutrophil cytoplasmic antibody was not detected. Serologic specimens also tested negative for cytomegalovirus, herpes simplex virus, Epstein-Barr virus, mycoplasma, hepatitis C virus (HCV) antibody, and hepatitis B virus surface (HBs) antigen. The tuberculin skin test was negative for the purified protein derivative. Although several small LNs swelling in the para-aortic and bilateral renal artery branching area were detected in an abdominal CT scan, any abnormal finding was not confirmed in FDG-PET. Chest CT showed no finding such as interstitial pneumonia. Systemic lymphadenopathy, polyclonal hypergammaglobulinemia associated with IgE and IgG4 elevation, hypocomplementemia, and renal dysfunction reminded us of development of IgG4 related disease, and echo-guided percutaneous kidney biopsy was performed on the 7th hospital day. Four out of 28 glomeruli showed global sclerosis, and 2 glomeruli collapsed with periglomerular fibrosis. The other glomeruli showed mild or no mesangial proliferative change. The biopsy revealed interstitial inflammatory cell infiltration, including infiltration of lymphocytes and plasma cells, and concurrent segmental glomerulonephritis (Figure 2A, B and 2C). Direct immunofluorescence microscopy revealed apparent positive staining with anti-human IgA (Figure 2D) and anti-human IgG antibodies in the mesangium, Complement 3 deposition was also recognized. Anti-human IgG4 antibody staining revealed many positive plasma cells in the interstitium (Figure 2E). The ratio of IgG4-positive plasma cells to IgG-positive plasma cells was more than 50% (data not shown). Electron micrograph revealed numerous electron-dense deposits in the mesangium. Subepithelial electron-dense deposit in the capillary wall was not detected (Figure 2F).

The patient was diagnosed with HSP nephritis that was complicated by IgG4-related TIN. The patient was treated with PSL (30 mg/day) for 14 days, followed by tapering of PSL. As a result of the treatment, the swelling of the LNs decreased, but the patient continued to have persistent hematoproteinuria.

## Discussion

HSP has been recognized as a distinct clinical condition. The syndrome is also referred to as anaphylactoid purpura and allergic purpura because of circumstantial evidence implicating hypersensitivity to bacteria or viruses as a possible cause. Histopathological examinations revealed that the cutaneous lesions result from leukocytoclastic vasculitis. Immunofluorescence studies have revealed immunoglobulin (Ig) and complement component deposits in the cutaneous blood vessels and kidney, but serum complement levels are usually normal. IgA is the most abundant and sometimes the only Ig found in the skin and kidney lesions. The morphologic and immunopathologic features are similar in HSP nephritis and IgA nephropathy (IgAN), which is characterized by various degrees of focal or diffuse mesangial proliferation, diffuse deposition of IgA in the mesangium, and electron-dense deposits in the mesangium [3].

It has became well known that the elevation of serum IgG4 concentration and abundant IgG4-positive plasma cell infiltration in the pancreas are characteristic findings in autoimmune pancreatitis (AIP) [4], and IgG4-related TIN is also considered to belong to the same disease spectrum. Accordingly, the concept of IgG4-related systemic disease have not been established [1,5-11], the patients with this diseases share many common features; (1) elevated serum IgG4 level, (2) abundant IgG4-positive plasma cell infiltration in the affected organs, and (3) marked improvement with corticosteroid therapy [5,7,9,10,12-16]. Our patient exhibited these 3 features. Further, immunohistological studies revealed IgG, IgA, and C3 deposition in the glomeruli resembling IgAN. Although IgA nephropathy associated with MPO-ANCA positive glomerulonephritis has also been reported previously [17,18], renal biopsy of this case did not show any finding of necrotizing or crescentic glomerulonephritis. This patient had symptoms of HSP systemically. Therefore we made a diagnosis of concomitant HSP nephritis and IgG4-related TIN. Recently, it has been reported that several IgG4-related TIN complicated with glomerular disease [9,19]. However, this may be the first case of IgG4-related TIN with HSP nephritis.

Allergy research has elucidated the relationship among IgG antibodies, allergens, and the IgG4 subclass in patients undergoing allergen-specific immunotherapy [20]^, ^[21], and it has been shown that extended and high-dose exposure to occupational or injected allergens can induce an increase in IgG and IgG4 antibodies and a decrease in IgE antibodies [22-24]. IgG4 is produced in response to repeated exposure to environmental antigens [25]^, ^[26]. Our patient had been experiencing relapsing HSP for 4 years, and this episode might indicate that he may have been repeatedly exposed to the allergen (Figure 1). Although the nature of the allergen that triggers HSP is unknown, the facts that amounts of IgG were extremely high at the point of purpura development indicate protection with the production of IgG4 might be induced by repetitive allergen exposures, and this hard protection may related with the development of IgG4 related TIN. Nevertheless, HSP developed 3 times, and therefore HSP nephritis might be complicated.

We described a rare case of HSP complicated by concurrent IgG4-related TIN. A biopsy of the collected specimens revealed IgG, IgA, and C3 deposition in the glomeruli and IgG4-producing plasma cell infiltration in the interstitium. We speculate that HSP resulting from repeated allergen exposure might induce the development of IgG4-related TIN and also HSP nephritis.

## Competing Interests

The authors declare that they have no competing interests.

## Authors' contributions

RT and YH provided clinical care, HN conceived the report, and SH performed all the immunochemistry. KM and TS participated in the design of this report. All authors have read and approved the final manuscript.

## Consent

Written informed consent was obtained from the patient for publication of this case report and accompanying images. A copy of the written consent is available for review by the Editor-in-Chief of this journal.
